# Associations between domains of physical literacy by weight status in 8- to 12-year-old Canadian children

**DOI:** 10.1186/s12889-018-5898-3

**Published:** 2018-10-02

**Authors:** Christine Delisle Nyström, Gregory Traversy, Joel D. Barnes, Jean-Philippe Chaput, Patricia E. Longmuir, Mark S. Tremblay

**Affiliations:** Healthy Active Living and Obesity (HALO) Research Group, Children’s Hospital of Eastern Ontario Research Institute, 401 Smyth Road, Ottawa, ON K1H 8L1 Canada

**Keywords:** Body mass index, Children, Daily behaviour, Knowledge and understanding, Motivation and confidence, Physical competence, Physical literacy, Waist circumference

## Abstract

**Background:**

The Canadian Assessment of Physical Literacy (CAPL) is divided into four domains (Physical Competence, Daily Behaviour, Motivation and Confidence, and Knowledge and Understanding) and provides a robust and comprehensive assessment of physical literacy. As weight status is known to influence health-related behaviours such as physical fitness and activity, it is important to investigate whether the associations between the domains of physical literacy vary among children of different weight status. The aim of this study was to determine the associations among the four domains of physical literacy stratified by weight status.

**Methods:**

Canadian children aged 8 to 12 years (*n* = 8343, 63.6% healthy-weight) completed the CAPL. Differences in domain scores and overall physical literacy score by weight status (children of healthy weight versus children with overweight/obese) were assessed using MANOVA (multivariate analysis of variance). Partial correlations between the four domains were calculated, adjusting for gender and age, and correlation coefficients of both weight status groups were compared using the Steiger test.

**Results:**

For all four domains as well as overall physical literacy, healthy-weight children had higher scores than their overweight/obese peers (Cohen’s *d* ranged from 0.05 to 0.44). Weak to moderate correlations were found between all of the domains for both groups. Correlation coefficients for Physical Competence and Daily Behaviour as well as for Physical Competence and Knowledge and Understanding were generally stronger in the healthy-weight children (*r* = 0.29 and 0.22, respectively) compared with the overweight/obese children (*r* = 0.23 and 0.17, respectively).

**Conclusions:**

All of the domains of the CAPL correlate positively with each other regardless of weight status, with a trend for these correlation coefficients to be slightly stronger in the healthy-weight children. The overall weak to moderate correlations between the domains in both groups suggest that the CAPL domains are not measuring the same constructs, thus providing support for CAPL’s psychometric architecture in both healthy-weight and overweight/obese children.

**Electronic supplementary material:**

The online version of this article (10.1186/s12889-018-5898-3) contains supplementary material, which is available to authorized users.

## Background

Childhood overweight and obesity represent a significant public health challenge globally [1]. Even though there is evidence that childhood obesity is stabilizing, levels are the highest they have ever been [2], with the prevalence of overweight and obesity in Canadian children aged 6 to 11 years estimated to be approximately 26% [3]. Childhood obesity is multifaceted; however, low levels of physical activity as well as high amounts of sedentary time are well-established correlates [4]. Physical literacy (PL) is defined as “the motivation, confidence, physical competence, knowledge and understanding to value and take responsibility for engagement in physical activities for life” [5]. The concept of PL has gained interest in recent years as an important construct that may help to explain why children may or may not engage in physical activity [6]. Obesity has been identified as an important correlate that significantly influences an individual’s level of PL [7]. Therefore, understanding how obesity impacts childhood PL may help children who are overweight or obese to live more active lives.

The Canadian Assessment of Physical Literacy (CAPL) was created to address the lack of objective PL data, and provides a robust and comprehensive aggregate assessment of PL. The overall aim of the CAPL is to provide a reliable, feasible, and valid instrument to assess PL in children [8–10]. In alignment with the Canadian consensus definition of PL [5], the four domains included within the CAPL are: Physical Competence, Daily Behaviour, Motivation and Confidence, and Knowledge and Understanding. Participants who complete the CAPL receive individual domain scores as well as an overall PL score [11]. There is a multitude of uses for the CAPL; these include but are not limited to: informing individual programs, providing evidence for resource allocation, influencing policy decisions, and providing national surveillance [10].

In order to ensure that the CAPL accurately assesses the various skills and behaviours included within it across all weight statuses, it is important to understand the associations between the individual domains for both healthy-weight and overweight/obese individuals. Furthermore, it has been suggested that PL is associated with weight status [7] and various behavioural, physical, social, and psychological factors [12]. Therefore, it is important to understand whether the individual domain scores assessed via the CAPL, and their associations between each other, vary between children of differing weight status.

Larouche et al. [13] investigated the associations between physical competence (i.e., physical fitness and motor skills) and daily physical activity, which is part of the Daily Behaviour domain, and found significant associations between the two domains. To date, no studies have investigated the correlations among all four domains of PL by weight status. A better understanding of these associations will provide important evidence as to whether the four domains of PL are related to each other and whether weight status influences these relationships. Therefore, the aim of this study was to determine the associations among the four domains of PL assessed via the CAPL in 8- to 12-year-old Canadian children stratified by weight status (i.e., healthy-weight versus overweight/obese).

## Methods

### Study design and participants

The Royal Bank of Canada (RBC) Learn to Play CAPL project was a nation-wide, cross-sectional study designed to assess the PL of Canadian children aged 8 to 12 years. Data were collected between February 2014 and February 2017 in 11 cities (seven provinces) across Canada. Participating cities included: Victoria, British Columbia; Lethbridge and Calgary, Alberta; Winnipeg, Manitoba; North Bay, Windsor, and Ottawa, Ontario; Trois-Rivières, Québec; Antigonish and Halifax, Nova Scotia; and Charlottetown, Prince Edward Island. In each testing site, a convenience sampling method was used to recruit participants, from a variety of settings (e.g., primary schools, after-school programs, and community centres) to obtain data for children from urban, suburban, and rural areas, and from various socioeconomic classes. Participants with missing data relevant to the analyses (*n* = 1545) as well as children who were classified as underweight (*n* = 130) were excluded from these analyses, which resulted in a final sample of 8343 children for the present analyses. The children included in these analyses did not differ from those excluded (based on missing data) with regard to demographic characteristics (e.g., gender, age, and body mass index [BMI]).

Parents or legal guardians provided written, informed consent and the children also provided assent to participate in this study. The CAPL project was approved first by the coordinating centre’s Research Ethics Board (Children’s Hospital of Eastern Ontario), and thereafter by each participating site’s institutional Research Ethics Board and participating school boards as required.

### CAPL protocol

The PL of all participants was evaluated following the published CAPL protocol [9, 14]. The CAPL manual [11] as well as other relevant material (e.g., training videos) are available online at https://www.capl-ecsfp.ca. The CAPL is made up of four domains (Physical Competence, Daily Behaviour, Knowledge and Understanding, and Motivation and Confidence), and provides domain scores as well as an overall PL score [9]. The Physical Competence and Daily Behaviour domains had maximum scores of 32 points each, whereas the Knowledge and Understanding and Motivation and Confidence domains had maximum scores of 18 points each. Thus, the maximum PL score was 100 points [9, 14]. A brief description of each of the four domains of PL is provided below, with more details provided elsewhere in this supplement [15].

#### Physical competence

This domain included objective measures of physical fitness, motor performance, and body composition. Physical fitness was assessed using: the Progressive Aerobic Cardiovascular Endurance Run (PACER) (cardiorespiratory fitness) [16], handgrip strength (muscular strength) [17], the abdominal plank test (muscular endurance) [18], and the sit-and-reach test (flexibility) [17]. Motor performance was evaluated using the Canadian Agility and Movement Skill Assessment (CAMSA) [19], and body composition was measured using BMI z-scores [20] as well as waist circumference [17]. The total number of available points for this domain was 32, with the PACER and CAMSA comprising of 8.4 points each; grip strength, plank, BMI z-score, and waist circumference comprising of 3.4 points each; and sit and reach comprising of 1.6 points [9].

#### Daily behaviour

The Daily Behaviour domain included objective and subjective measures of physical activity and sedentary behaviour. Physical activity was measured objectively using pedometers (YamaxDigiWalker SW-200, Yamax Corporation, Tokyo, Japan) for seven consecutive days, in which the participants were required to wear them during waking hours. Two questionnaire items were used to assess the number of days the children engaged in at least 60 min of moderate to vigorous physical activity (MVPA) in the past week as well as to assess their daily screen time habits on both weekdays and weekend days [11]. The majority of this domain score was from the objectively measured physical activity (21 of 32 available points), while the subjectively measured sedentary and MVPA times were comprised of 8 and 3 points, respectively [9].

#### Motivation and confidence

This domain was assessed using a questionnaire, with items extracted from published instruments, to evaluate the children’s motivation and confidence to be physically active. The Children’s Self-Perception of Adequacy in and Predilection for Physical Activity (CSAPPA) sub-scales were used to assess adequacy and predilection for physical activity [21], which made up the majority of the domain score (12 of 18 available points). A ratio of perceived benefits and barriers of physical activity [22] as well as questions regarding how their skills and activity levels compared to their peers [9] made up the remainder of the domain score (4 and 2 points, respectively).

#### Knowledge and understanding

This domain was evaluated using a questionnaire to assess the participant’s knowledge and understanding of: recommendations for physical activity and sedentary behaviour, health-related terminology, how to improve motor and fitness skills, and the utilization of safety equipment in daily life. All questions were equally weighted and were created so that they were applicable for children from 8 to 12 years of age, with this domain comprising of 18 points [23].

### Analytical strategy

#### Calculation of domain scores and overall PL score

The calculations for the scores for all domains are described in detail in the CAPL manual [11]. The Physical Competence domain had to be recalculated by removing the body composition variables (i.e., BMI z-score and waist circumference), as all analyses were stratified by these variables. Due to the strong correlation between BMI and waist circumference (*r* = 0.89, *p* <  0.001), both indices were removed from the Physical Competence domain, regardless of the variable that was being used for stratification. The Physical Competence domain was then re-weighted and the overall PL score was re-calculated to account for the new Physical Competence score. From this point and throughout the rest of the manuscript, Physical Competence will be referred to as modified Physical Competence.

#### Weight status categories

The BMI for each child was calculated as weight (kg) divided by height (m) squared. Children were categorized into weight status categories using BMI z-scores based on the criteria of the World Health Organization (WHO), which are widely accepted [20]. Using these WHO standards, a child with a BMI z-score > 1 and ≤ 2 standard deviations was considered overweight, while a child with a BMI z-score > 2 standard deviations was considered obese [20]. A BMI z-score of < − 2 standard deviations was considered underweight; however, due to the small number of children in this group (*n* = 130), they were excluded from the analyses. Finally, the children who were classified as overweight and obese were grouped together to maximize power.

Waist circumference was measured by marking the child’s hip bone on both sides and placing the tape measure around the child covering the two marks. The child was then told to breathe in and then out and relax while the measurement was taken. Children were grouped into two categories based on waist circumference using the Centers for Disease Control and Prevention reference values, which were based on the National Health and Nutrition Examination Surveys from 2011 to 2012 and 2013–2014 [24]. Using sex- and age-specific percentiles, children less than or equal to the 85th percentile were defined as healthy-weight, whereas children greater than the 85th percentile were grouped into the overweight/obese category.

### Statistical analysis

Values are presented as means and standard deviations. Differences between groups (healthy-weight versus overweight/obese) for demographic characteristics, domain scores, and the overall PL score were determined using multivariate analysis of variance (MANOVA). Categorical demographic characteristics were compared using the chi-squared test.

Partial correlations were used to assess the associations between each of the domain scores of PL separately, while controlling for age and gender (as these have been shown to influence some CAPL domain scores as well as the overall PL score) [9]. Correlation coefficients were classified as weak (0.1 ≤ *r* <  0.3), moderate (0.3 ≤ *r* <  0.5), or strong (*r* ≥ 0.5) [25]. Analyses were conducted separately for participants categorized as healthy weight or overweight/obese. The Steiger test was used to determine if the correlation coefficients between the domains of PL differed significantly between children who were categorized as healthy- weight and those categorized as overweight/obese. Sensitivity analyses were also conducted running the same analyses except stratifying by waist circumference as well as comparing only healthy-weight and obese children. Effect sizes were examined using Cohen’s *d* method, reflecting the magnitude of the difference between the two groups. Effect sizes were considered negligible if < 0.2, small if between 0.2 and 0.5, moderate if between 0.5 and 0.8, and important if > 0.8. Statistical analyses were conducted using RStudio version 1.1.383 (RStudio, Boston, MA, USA). In addition to the use of base R, the “psych” (cran.r-project.org/web/packages/psych/psych.pdf), “ppcor” (cran.r-project.org/web/packages/ppcor/ppcor.pdf), “boot” (cran.r-project.org/web/packages/boot/boot.pdf), and “lsr” (cran.r-project.org/web/packages/lsr/lsr.pdf) packages were also loaded for use in the analyses. All analyses were two-sided with a 5% level of significance.

## Results

Table 1 presents the descriptive characteristics of the participants stratified by BMI category. Out of the total sample (*n* = 8343) 36.4% of the children were classified as overweight/obese. Furthermore, of the 3036 children classified as overweight/obese, 58.7% (*n* = 1782) were classified as overweight and 41.3% (*n* = 1254) were classified as obese.

**Table 1:** Descriptive characteristics^a^ of participants stratified by weight status^b^

	Total sample (*n* = 8343)	Healthy- weight (*n* = 5307)	Overweight/obese (*n* = 3036)	Difference *p* value^c^	Cohen’s *d* effect size^d^
Gender (girl)	4183 (50.1%)	2715 (51.2%)	1468 (48.4%)	0.007	–
Age (years)	10.6 ± 1.2	10.6 ± 1.2	10.6 ± 1.1	0.080	0.04
Height (cm)	144.2 ± 9.9	142.7 ± 9.7	146.8 ± 9.7	< 0.001	0.42
Weight (kg)	40.1 ± 11.4	34.6 ± 6.6	49.8 ± 11.6	< 0.001	1.75
BMI (kg/m^2^)	19.0 ± 3.8	16.8 ± 1.5	22.9 ± 3.4	< 0.001	2.58
Waist circumference (cm)^e^	67.4 ± 10.7	61.7 ± 5.6	77.3 ± 10.3	< 0.001	2.05

*BMI* body mass index

^a^Data are presented as mean ± standard deviation or n (%)

^b^Body weight status was defined according to the World Health Organization reference data [20]

^c^*p* values were assessed using multivariate analysis of variance (continuous variables) and a chi-squared test (categorical variable)

^d^Effect sizes are considered negligible if < 0.2, small if between 0.2 and 0.5, moderate if between 0.5 and 0.8, and important if > 0.8

^e^Sample sizes for waist circumference were *n* = 5242 and *n* = 2976 for healthy-weight and overweight/obese, respectively

The individual domain scores as well as the overall PL score stratified by BMI category are displayed in Table 2. For all four domain scores as well as the overall PL score, children who were classified as healthy-weight had higher scores than their overweight/obese peers (range for *p*: < 0.001 to 0.024). However, effect sizes for most of these comparisons were considered negligible (all < 0.2), except for modified Physical Competence and overall PL (Cohen’s *d* = 0.44 and 0.30, respectively).

**Table 2:** Physical literacy scores^a^ stratified by weight status^b^ (n = 8343)

	Healthy-weight (n = 5307)	Overweight/obese (*n* = 3036)	Difference *p* value^c^	Cohen’s *d* effect size^d^
Modified Physical Competence	19.2 ± 4.7	17.2 ± 4.1	< 0.001	0.44
Daily Behaviour	18.9 ± 7.4	17.9 ± 7.7	< 0.001	0.13
Motivation and Confidence	12.7 ± 2.7	12.3 ± 2.8	< 0.001	0.15
Knowledge and Understanding	12.1 ± 2.7	12.0 ± 2.8	0.024	0.05
Total CAPL score	62.8 ± 12.1	59.3 ± 11.6	< 0.001	0.30

*CAPL* Canadian Assessment of Physical Literacy

^a^Data are presented as means ± standard deviation

^b^Body weight status was defined according to the World Health Organization reference data [20]

^c^Physical literacy scores were compared using multivariate analysis of variance

^d^Effect sizes are considered negligible if < 0.2, small if between 0.2 and 0.5, moderate if between 0.5 and 0.8, and important if > 0.8

In the healthy-weight group, positive associations were found among all PL domains (Table 3). For modified Physical Competence, there was a weak correlation with Daily Behaviour and Knowledge and Understanding, whereas there was a moderate correlation with Motivation and Confidence. There was a moderate correlation between Daily Behaviour and Motivation and Confidence, and weak correlations between Daily Behaviour and Knowledge and Understanding, as well as between Motivation and Confidence and Knowledge and Understanding. Table 4 presents the partial correlations for the domains of PL for the children classified as overweight/obese; these yielded similar results to the healthy-weight children. The correlations between modified Physical Competence and Daily Behaviour, as well as between modified Physical Competence and Knowledge and Understanding, were significantly higher in the healthy-weight children compared to the overweight/obese children (Z = 2.69 and 2.65, respectively; both *p* <  0.01). However, the difference in the correlation coefficients was small.

**Table 3:** Partial correlations^a^ and 95% confidence intervals between physical literacy domain scores in healthy-weight children^b^ (controlled for age and gender) (n = 5307)

	Modified Physical Competence	Daily Behaviour	Motivation and Confidence	Knowledge and Understanding
Modified Physical Competence	1	–	–	–
Daily Behaviour	0.29* (0.27–0.32)	1	–	–
Motivation and Confidence	0.42* (0.40–0.44)	0.35* (0.33–0.38)	1	–
Knowledge and Understanding	0.22* (0.20–0.25)	0.10* (0.08–0.13)	0.21* (0.18–0.23)	1

^a^Correlation coefficients were classified as weak (0.1 ≤ *r* < 0.3), moderate (0.3 ≤ *r* < 0.5), or strong (*r* ≥ 0.5) [25]

^b^Body weight status was defined according to the World Health Organization reference data [20]

*Significant correlation coefficients, *p* < 0.001

**Table 4:** Partial correlations^a^ and 95% confidence intervals between physical literacy domain scores in overweight/obese children^b^ (controlled for age and gender) (n = 3036)

	Modified Physical Competence	Daily Behaviour	Motivation and Confidence	Knowledge and Understanding
Modified Physical Competence	1	–	–	–
Daily Behaviour	0.23* (0.20–0.27)	1	–	–
Motivation and Confidence	0.39* (0.37–0.42)	0.34* (0.31–0.38)	1	–
Knowledge and Understanding	0.17* (0.13–0.20)	0.07* (0.03–0.11)	0.17* (0.13–0.20)	–

^a^Correlation coefficients were classified as weak (0.1 ≤ *r* < 0.3), moderate (0.3 ≤ *r* < 0.5), or strong (*r* ≥ 0.5) [25]

^b^Body weight status was defined according to the World Health Organization reference data [20]

*Significant correlation coefficients, *p* < 0.001

In sensitivity analyses, we conducted the same analyses, except the analyses were stratified by waist circumference (≤ 85th percentile and > 85th percentile) (see Additional file 1), and we compared only healthy-weight and obese children as classified by BMI (see Additional file 2). The effect sizes were slightly larger for both sensitivity analyses when stratifying by waist circumference or healthy-weight versus obese; however, the effect sizes were still classified as weak to moderate. The largest differences were observed for modified Physical Competence and overall PL. With regards to the associations between the domains of PL, similar results were obtained for both sensitivity analyses in comparison to the original analysis.

## Discussion

The aim of this study was to examine the associations among the four domains of PL assessed using the CAPL, stratified by weight status (i.e., healthy-weight versus overweight/obese). In general, there was a tendency for the correlations to be slightly stronger in the healthy-weight group than the overweight/obese group. However, only two correlations differed significantly between the groups; those were between modified Physical Competence and Daily Behaviour, and between modified Physical Competence and Knowledge and Understanding. Comparable moderate correlations were found for the healthy-weight and overweight/obese groups between modified Physical Competence and Motivation and Confidence (*r* = 0.42 and 0.39, respectively) as well as between Daily Behaviour and Motivation and Confidence (*r* = 0.35 and 0.34, respectively). Therefore, these results suggest that motivation and confidence are important correlates of modified Physical Competence and Daily Behaviour, irrespective of weight status.

In this study, we found that healthy-weight children had significantly higher scores for modified Physical Competence and Daily Behaviour than overweight/obese children, which is in line with previous research. For instance, Fairclough et al. [26] found that overweight/obese children undertook less light physical activity and MVPA than their healthy-weight counterparts. Furthermore, it has been shown that healthy-weight adolescents scored significantly higher on tests of aerobic fitness, motor fitness, and lower body muscular strength than their overweight/obese peers [27]. More specifically, in children it has been found that overweight children complete less laps in the 20-m shuttle run compared to healthy-weight children (mean ± standard deviation: 16.2 ± 8.1 and 32.1 ± 13.8 laps, respectively; Cohen’s *d* = 1.4) [26]. Finally, there is evidence that a higher BMI has a negative relationship with certain fundamental movement skills (e.g., skill composite and motor coordination) [28].

Moderate correlations were observed between the Daily Behaviour domain and the Motivation and Confidence domain, with no significant differences found between healthy-weight and overweight/obese participants. De Bourdeaudhuij et al. [29] investigated physical activity and psychosocial correlates in children and adolescents and found that in comparison to healthy-weight participants, overweight participants had fewer favourable psychosocial correlates in relation with physical activity (*p* <  0.001). However, similar to our results, they found that the strength of the associations between physical activity and the investigated psychosocial factors to be similar between both groups (e.g., general attitudes of fun: *r* = 0.41 and 0.39 and perceived benefits from pleasure: *r* = 0.28 and 0.24, for healthy-weight and overweight participants, respectively). Moderate correlations were also observed between modified Physical Competence and Motivation and Confidence, with no differences observed between the healthy-weight and overweight/obese children. Our results are supported by a study by Cairney et al. [30], who found that greater perceived adequacy regarding physical activity and higher predilection to select physical over sedentary activities (as assessed using CSAPPA) were independently related with a stronger performance in the 20-m shuttle run.

The weak associations observed between Knowledge and Understanding and the other domains is not necessarily surprising, as it has been suggested that although knowledge is an important element for promoting physical activity, it is not enough in itself to change behaviour [31]. While a recent prospective, observational study in Chinese children observed that children who became cognizant of the relationship between physical activity and weight became more active, these results need to be interpreted cautiously as this study used subjective measures to assess physical activity [32].

Additionally, we found a significant difference between the correlation coefficients between healthy-weight and overweight/obese children for modified Physical Competence and Knowledge and Understanding (*r* = 0.22 and 0.17, for healthy-weight and overweight/obese children, respectively). A possible explanation for this finding could be that the healthy-weight individuals who have higher levels of physical competence than their overweight/obese peers may also have greater knowledge due to a common source (e.g., higher levels of participation in physical education or in organized sport); however, this is purely speculative at this stage. Due to the lack of research in this area, further studies are warranted.

This study is strengthened through the use of a reliable and valid protocol to assess PL (i.e., the CAPL) [9, 14, 18, 19], the large sample size, and the standardized methods used to collect data. Limitations of the present study include the cross-sectional design (which limits the ability to make causal inferences), as well as the use of pedometers to measure physical activity (as they cannot differentiate between different intensity levels). Additionally, the children were recruited using convenience sampling and therefore might not be representative of 8- to 12-year-old Canadian children in general. For instance, the prevalence of overweight/obesity in the participating children (36%) was higher than the prevalence of overweight/obesity in the general population (26%) [3]. Finally, although the correlations were controlled by age and gender, the CAPL does not include a measure of maturation, which could possibly influence the results.

Due to the fact that the CAPL domains measure factors that may be related to one another (e.g., physical fitness and overall physical activity), there may be concerns that the domains are overlapping. However, a large proportion of the variance in the domain scores was not explained by the correlations examined in this study (*r*^2^ = 0.005 to 0.176). Furthermore, the majority of the associations were of low to moderate strength, which lends support to the psychometric architecture of the CAPL in both healthy-weight and overweight children as well as the relatively unique contribution each domain makes to the composite PL score. If strong associations were found, it might have suggested that the domains are duplicating each other and that the CAPL should therefore be revised. However, the results of this study indicate that this is not the case.

Beyond providing validity evidence for the CAPL for children who are overweight/obese as well as children who are healthy-weight, the results of this study have broader implications for PL. The associations between most of the domains of PL were stable across children of different weight status. Even though significant differences were found between the healthy-weight and overweight/obese children for the associations between modified Physical Competence and Daily Behaviour, and between modified Physical Competence and Knowledge and Understanding, for both groups the correlations were classified as weak. This suggests that future interventions aimed at improving PL probably do not need to be tailored based on weight status, although longitudinal studies are needed to confirm these conclusions. Finally, the results indicate that future PL interventions should target all domains of PL, as there does not seem to be one domain in particular that could be specifically targeted in order to improve the scores in the other domains.

## Conclusions

Healthy-weight children scored higher in all four domains and overall PL in comparison to overweight/obese children, although the differences were small to negligible. Weak to moderate correlations among all domains were observed for both groups. The overall weak to moderate correlations found between the domains suggest that the four domains of CAPL are not measuring the same constructs, thus providing support for CAPL’s psychometric architecture in both healthy-weight and overweight/obese children.

## Additional files

Additional file 1: Physical literacy scores stratified by waist circumference, and partial correlations between physical literacy domain scores for children stratified by waist circumference. (DOCX 20 kb)

Additional file 2: Physical literacy scores comparing healthy-weight and obese children as classified by BMI, and partial correlations between physical literacy domain scores for children classified as obese. (DOCX 17 kb)

## Abbreviations

BMI: Body mass index
CAMSA: Canadian Agility and Movement Skill Assessment
CAPL: Canadian Assessment of Physical Literacy
CSAPPA: Children’s Self-Perception of Adequacy in and Predilection for Physical Activity
MANOVA: Multivariate analysis of variance
MVPA: Moderate-to-vigorous physical activity
PACER: Progressive Aerobic Cardiovascular Endurance Run
PL: Physical literacy
RBC: Royal Bank of Canada
WHO: World Health Organization

## References

[1] World Health Organization. The commision on ending childhood obesity. World Health Organization. 2017. www.who.int/end-childhood-obesity/publications/echo-plan-executive-summary/en. Accessed 6 Feb 2018.

[2] Rokholm B, Baker JL, Sorensen TI. The levelling off of the obesity epidemic since the year 1999 – a review of evidence and perspectives. Obes Rev. 2010;11:835–46. 10.1186/s12889-018-5903-x 10.1111/j.1467-789X.2010.00810.x 20973911

[3] Rao DP, Kropac E, Do MT, Roberts KC, Jayaraman GC (2016). Childhood overweight and obesity trends in Canada. Health Promot Chronic Dis Prev Can.

[4] Prentice-Dunn H, Prentice-Dunn S (2012). Physical activity, sedentary behaviour, and childhood obesity: a review of cross-sectional studies. Psychol Health Med.

[5] Tremblay MS, Costas-Bradstreet C, Barnes JD, Bartlett B, Dampier D, Lalonde C, et al. Canada’s Physical Literacy Consensus Statement: process and outcome. BMC Public Health. 2018;18(Suppl 2) 10.1186/s12889-018-5903-x. 10.1186/s12889-018-5903-x PMC6167775 30285701

[6] Whitehead M (2010). Physical literacy: throughout the lifecourse.

[7] Gately P, Whitehead M (2010). Physical literacy and obesity. Physical literacy: throughout the lifecourse.

[8] Longmuir P. Understanding the physical literacy journey of children: the Canadian assessment of physical literacy. Bulletin of the International Council of Sport Science and Physical Education ICSSPE. 2013;65:1–6.

[9] Longmuir PE, Boyer C, Lloyd M, Yang Y, Boiarskaia E, Zhu W (2015). The Canadian assessment of physical literacy: methods for children in grades 4 to 6 (8 to 12 years). BMC Public Health.

[10] Tremblay MS, Lloyd M (2010). Physical literacy measurement – the missing piece. PHE Journal.

[11] Healthy Active Living and Obesity Research Group. Canadian assessment of physical literacy: manual for test administration. Healthy Active Living and Obesity Research Group. 2013. https://www.capl-ecsfp.ca/capl-manual/. Accessed 6 Feb 2018.

[12] Edwards LC, Bryant AS, Keegan RJ, Morgan K, Jones AM (2017). Definitions, foundations and associations of physical literacy: a systematic review. Sports Med.

[13] Larouche R, Boyer C, Tremblay MS, Longmuir P (2014). Physical fitness, motor skill, and physical activity relationships in grade 4 to 6 children. Appl Physiol Nutr Metab.

[14] Francis CE, Longmuir PE, Boyer C, Andersen LB, Barnes JD, Boiarskaia E (2016). The Canadian assessment of physical literacy: development of a model of children’s capacity for a healthy, active lifestyle through a Delphi process. J Phys Act Health.

[15] Tremblay MS, Longmuir PE, Barnes JD, Belanger K, Anderson KD, Bruner B, et al. Physical literacy levels of Canadian children aged 8–12 years: descriptive and normative results from the RBC Learn to Play-CAPL project. BMC Public Health. 2018;18(Suppl 2) 10.1186/s12889-018-5891-x. 10.1186/s12889-018-5891-x PMC6167776 30285693

[16] Carrel AL, Bowser J, White D, Moberg DP, Weaver B, Hisgen J (2012). Standardized childhood fitness percentiles derived from school-based testing. J Pediatr.

[17] CSEP (2013). Canadian Society for Exercise Physiology – physical activity training for health.

[18] Boyer C, Tremblay MS, Saunders TJ, McFarlane A, Borghese M, Lloyd M (2013). Feasibility, validity and reliability of the plank isometric hold as a field-based assessment of torso muscular endurance for children 8-12 years of age. Pediatr Exerc Sci.

[19] Longmuir PE, Boyer C, Lloyd M, Borghese MM, Knight E, Saunders TJ (2017). Canadian agility and movement skill assessment (CAMSA): validity, objectivity, and reliability evidence for children 8-12 years of age. J Sport and Health Sci.

[20] World Health Organization. Growth reference 5–19 years. World Health Organization. 2007. www.who.int/growthref/who2007_bmi_for_age/en. Accessed 6 Feb 2018.

[21] Hay JA (1992). Adequacy in and predilection for physical activity in children. Clin J Sport Med.

[22] Garcia AW, Broda MA, Frenn M, Coviak C, Pender NJ, Ronis DL (1995). Gender and developmental differences in exercise beliefs among youth and prediction of their exercise behaviour. J Sch Health.

[23] Longmuir PE, Woodruff SJ, Boyer C, Lloyd M, Tremblay MS. Physical Literacy Knowledge Questionnaire: feasibility, validity, and reliability for Canadian children aged 8 to 12 years. BMC Public Health. 2018;18(Suppl 2) 10.1186/s12889-018-5890-y. 10.1186/s12889-018-5890-y PMC6167766 30285679

[24] Fryar CD, Gu Q, Ogden CL, Flegal KM. Anthropometric reference data for children and adults: United States, 2011–2014. Vital Health Stat. 2016;3:1–46. 28437242

[25] Kenny DA (1987). Statistics for the social and behavioural sciences.

[26] Fairclough SJ, Dumuid D, Taylor S, Curry W, McGrane B, Stratton G (2017). Fitness, fatness and the reallocation of time between children’s daily movement behaviours: an analysis of compositional data. Int J Behav Nutr Phys Act.

[27] Izquierdo-Gomez R, Martinez-Gomez D, Fernhall B, Sanz A, Veiga OL (2016). The role of fatness on physical fitness in adolescents with and without Down syndrome: the UP&DOWN study. Int J Obes.

[28] Barnett LM, Lai SK, Veldman SLC, Hardy LL, Cliff DP, Morgan PJ (2016). Correlates of gross motor competence in children and adolescents: a systematic review and meta-analysis. Sports Med.

[29] De Bourdeaudhuij I, Lefevre J, Deforche B, Wijndaele K, Matton L, Philippaerts R (2005). Physical activity and psychosocial correlates in normal weight and overweight 11 to 19 year olds. Obes Res.

[30] Cairney J, Hay JA, Faught BE, Leger L, Mathers B (2008). Generalized self-efficacy and performance on the 20-metre shuttle run in children. Am J Hum Biol.

[31] Morrow JR, Krzewinski-Malone JA, Jackson AW, Bungum TJ, FitzGerald SJ (2004). American adults’ knowledge of exercise recommendations. Res Q Exerc Sport.

[32] Xu F, Wang X, Xiang D, Wang Z, Ye Q, Ware RS (2017). Awareness of knowledge and practice regarding physical activity: a population-based prospective, observational study among students in Nanjing. China PloS One.

