# Supplementary material for: Associations between domains of physical literacy by weight status in 8- to 12-year-old Canadian children
Source: BMC Public Health. 2018 Oct 2;18(Suppl 2):1043. doi: 10.1186/s12889-018-5898-3 (PMC6167768; doi:10.1186/s12889-018-5898-3)
Supplement: Supplementary file 1 — Physical literacy scores stratified by waist circumference, and partial correlations between physical literacy domain scores for children stratified by waist circumference. (DOCX 20 kb) [file 12889_2018_5898_MOESM1_ESM.docx]

**Table S1a.** Physical literacy scores^a^ stratified by waist circumference^b^ (n = 8218).

|  | ≤ 85^th^ percentile  (n = 7492) | > 85^th^ percentile  (n = 726) | Difference  *p-*value^c^ | Cohen’s *d*^d^ |
| --- | --- | --- | --- | --- |
| Modified Physical Competence | 18.7 ± 4.6 | 15.7 ± 3.5 | < 0.001 | 0.68 |
| Daily Behaviour | 18.7 ± 7.5 | 16.2 ± 7.7 | < 0.001 | 0.34 |
| Motivation and Confidence | 12.6 ± 2.7 | 11.8 ± 2.8 | < 0.001 | 0.30 |
| Knowledge and Understanding | 12.1 ± 2.7 | 11.6 ± 2.8 | < 0.001 | 0.17 |
| Total CAPL score | 62.1 ± 11.9 | 55.3 ± 10.8 | < 0.001 | 0.58 |

^a^ Data are presented as means ± standard deviation.

^b^ Waist circumference was defined according to Fryar et al. [24].

^c^ Physical literacy scores were compared using multivariate analysis of variance.

^d^ Effect sizes are considered negligible if < 0.2, small if between 0.2 and 0.5, moderate if between 0.5 and 0.8, and important if > 0.8.

CAPL: Canadian Assessment of Physical Literacy

**Table S1b.** Partial correlations^a^ and 95% confidence intervals between physical literacy domain scores in children with a waist circumference ≤ 85^th^ percentile^b^ (controlled for age and gender) (n = 7492).

|  | Modified Physical Competence | Daily Behaviour | Motivation and Confidence | Knowledge and Understanding |
| --- | --- | --- | --- | --- |
| Modified Physical Competence | 1 | - | - | - |
| Daily Behaviour | 0.27*  (0.25-0.29) | 1 | - | - |
| Motivation and Confidence | 0.41*  (0.39-0.43) | 0.35*  (0.33-0.37) | 1 | - |
| Knowledge and Understanding | 0.21*  (0.18-0.23) | 0.09*  (0.07-0.11) | 0.20*  (0.17-0.22) | 1 |

^a^ Correlation coefficients were classified as weak (0.1 ≤ *r* < 0.3), moderate (0.3 ≤ *r* < 0.5), or strong (*r* ≥ 0.5) [25].

^b^ Waist circumference was defined according to Fryar et al. [24].

*Significant correlations, *p* < 0.001

**Table S1c.** Partial correlations^a^ and 95% confidence intervals between physical literacy domain scores in children with a waist circumference > 85^th^ percentile^b^ (controlled for age and gender) (n = 726).

|  | Modified Physical Competence | Daily Behaviour | Motivation and Confidence | Knowledge and Understanding |
| --- | --- | --- | --- | --- |
| Modified Physical Competence | 1 | - | - | - |
| Daily Behaviour | 0.14**  (0.06-0.21) | 1 | - | - |
| Motivation and Confidence | 0.32*  (0.26-0.39) | 0.31*  (0.25-0.38) | 1 | - |
| Knowledge and Understanding | 0.12**  (0.05-0.20) | -0.03  (-0.04-0.11) | 0.16*  (0.09-0.23) | - |

^a^ Correlation coefficients were classified as weak (0.1 ≤ *r* < 0.3), moderate (0.3 ≤ *r* < 0.5), or strong (*r* ≥ 0.5) [25].

^b^ Waist circumference was defined according to Fryar et al. [24].

*Significant correlations, *p* < 0.001

**Significant correlations, *p* < 0.05
