# Supplementary material for: Associations between domains of physical literacy by weight status in 8- to 12-year-old Canadian children
Source: BMC Public Health. 2018 Oct 2;18(Suppl 2):1043. doi: 10.1186/s12889-018-5898-3 (PMC6167768; doi:10.1186/s12889-018-5898-3)
Supplement: Supplementary file 2 — Physical literacy scores comparing healthy-weight and obese children as classified by BMI, and partial correlations between physical literacy domain scores for children classified as obese. (DOCX 17 kb) [file 12889_2018_5898_MOESM2_ESM.docx]

**Table S2a.** Physical literacy scores^a^ stratified by weight status^b^ (n = 6561).

|  | Healthy-weight  (n = 5307) | Obese  (n = 1254) | Difference  *p-*value^c^ | Cohen’s *d*^d^ |
| --- | --- | --- | --- | --- |
| Modified Physical Competence | 19.2 ± 4.7 | 16.2 ± 3.6 | <0.001 | 0.66 |
| Daily Behaviour | 18.9 ± 7.4 | 17.2 ± 7.8 | <0.001 | 0.22 |
| Motivation and Confidence | 12.7 ± 2.7 | 12.1 ± 2.8 | <0.001 | 0.22 |
| Knowledge and Understanding | 12.1 ± 2.7 | 11.6 ± 2.8 | <0.001 | 0.17 |
| Total CAPL score | 62.8 ± 12.1 | 57.1 ± 11.1 | <0.001 | 0.48 |

^a^ Data are presented as means ± standard deviation.

^b^ Body weight status was defined according to the World Health Organization reference data [20].

^c^ Physical literacy scores were compared using multivariate analysis of variance.

^d^ Effect sizes are considered negligible if < 0.2, small if between 0.2 and 0.5, moderate if between 0.5 and 0.8, and important if > 0.8.

CAPL: Canadian Assessment of Physical Literacy

**Table S2b.** Partial correlations^a^ and 95% confidence intervals between physical literacy domain scores in children classified as obese^b^ (controlled for age and gender) (n = 1254).

|  | Modified Physical Competence | Daily Behaviour | Motivation and Confidence | Knowledge and Understanding |
| --- | --- | --- | --- | --- |
| Modified Physical Competence | 1 | - | - | - |
| Daily Behaviour | 0.21*  (0.16-0.26) | 1 | - | - |
| Motivation and Confidence | 0.38*  (0.33-0.43) | 0.34*  (0.29-0.39) | 1 | - |
| Knowledge and Understanding | 0.11*  (0.06-0.17) | 0.04  (-0.02-0.09) | 0.11*  (0.06-0.17) | - |

^a^ Correlation coefficients were classified as weak (0.1 ≤ *r* < 0.3), moderate (0.3 ≤ *r* < 0.5), or strong (*r* ≥ 0.5) [25].

^b^ Body weight status was defined according to the World Health Organization reference data [20].

*Significant correlations, *p* <0.001
